# Undertriage of major trauma patients at a university hospital: a retrospective cohort study

**DOI:** 10.1186/s13049-018-0524-z

**Published:** 2018-08-14

**Authors:** Terje Nordgarden, Peter Odland, Anne Berit Guttormsen, Kristina Stølen Ugelvik

**Affiliations:** 1 Faculty of Medicine, University of Bergen, Haukelandsveien 28, 5009 Bergen, Norway; 2 Department of Clinical Medicine 1, Jonas Lies vei 65, 5021 Bergen, Norway; 3 Department of Anaesthesia and Intensive Care, Haukeland University Hospital, Jonas Lies vei 65, 5021 Bergen, Norway; 4 Regional Trauma Center, Surgical Department, Haukeland University Hospital, Jonas Lies vei 65, 5021 Bergen, Norway

**Keywords:** American College of Surgeons, Committee on trauma, Guidelines for Field Triage of Injured Patients, Haukeland University Hospital, National Trauma Plan, Norway, Trauma, Trauma team, Undertriage

## Abstract

**Background:**

Studies show increased mortality among severely injured patients not met by trauma team. Proper triage is important to ensure that all severely injured patients receive vital trauma care. In 2017 a new national trauma plan was implemented in Norway, which recommended the use of a modified version of *“Guidelines for Field Triage of Injured Patients”* to identify severely injured patients.

**Methods:**

A retrospective study of 30,444 patients admitted to Haukeland University Hospital in 2013, with ICD-10 injury codes upon discharge. The exclusion criteria were department affiliation considered irrelevant when identifying trauma, patients with injuries that resulted in Injury Severity Score < 15, patients that did receive trauma team, and patients admitted > 24 h after time of injury. Information from patient records of every severely injured patient admitted in 2013 was obtained in order to investigate the sensitivity of the new guidelines.

**Results:**

Trauma team activation was performed in 369 admissions and 85 patients were identified as major trauma. Ten severely injured patients did not receive trauma team resuscitation, resulting in an undertriage of 10.5%. Nine out of ten patients were men, median age 54 years. Five patients were 60 years or older. All of the undertriaged patients experienced fall from low height (< 4 m). Traumatic brain injury was seen in six patients. Six patients had a Glasgow Coma Scale score ≤ 13. The new trauma activation guidelines had a sensitivity of 95.0% in our 2013 trauma population. The degree of undertriage could have been reduced to 4.0% had the guidelines been implemented and correctly applied.

**Conclusions:**

The rate of undertriage at Haukeland University Hospital in 2013 was above the recommendations of less than 5%. Use of the new trauma guidelines showed increased triage precision in the present trauma population.

## Background

Studies show increased survival among severely injured patients met by a dedicated trauma team [1–6] and that implementation of trauma centers improve outcome in trauma patients [1, 7–13]. Trauma centers require a vast amount of human and financial resources to function as intended [14, 15]. In order to justify the expense, it is essential to maintain a reliable system able to identify patients with severe injury, while excluding non- and minor injuries.

Early trauma evaluation can be a challenge. In light of its dynamic nature, the wide range of possible injuries and the limited tools at disposal, occasional mistriage is to be expected. Mistriage is divided into under- and overtriage: Undertriage is defined as the proportion of severely injured patients not managed by a dedicated trauma team, while overtriage is the proportion of patients not severely injured but still receiving such care. The degree of undertriage is an indicator of the sensitivity of the trauma system. Overtriage is unfortunate as it is costly and exhausts human and financial recourses [16, 17]. Undertriage of less than 5% and overtriage of 25–30% is deemed acceptable according to the American College of Surgeons, Committee on Trauma [18].

**Fig. 1:**
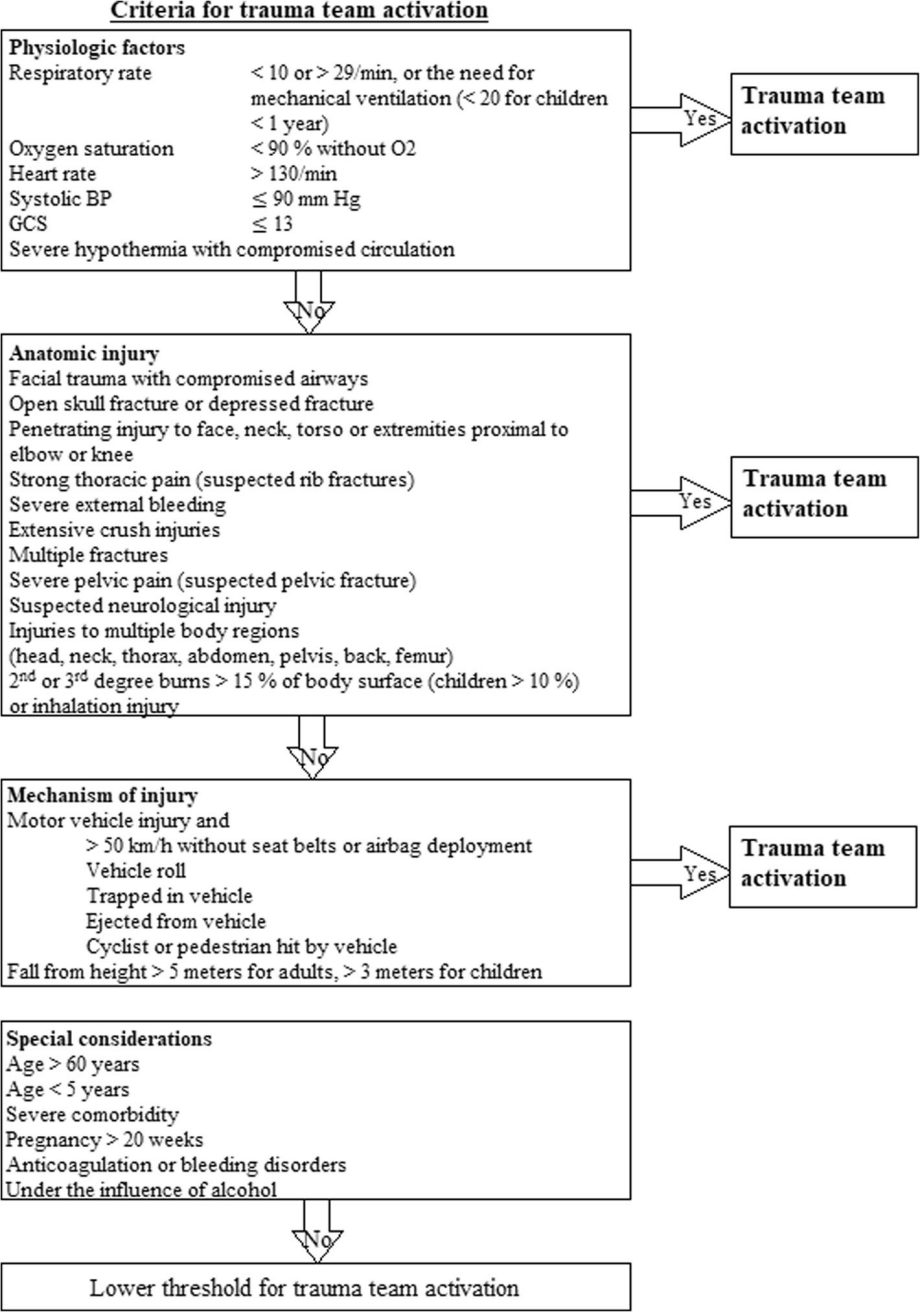
Trauma team activation criteria

**Fig. 2:**
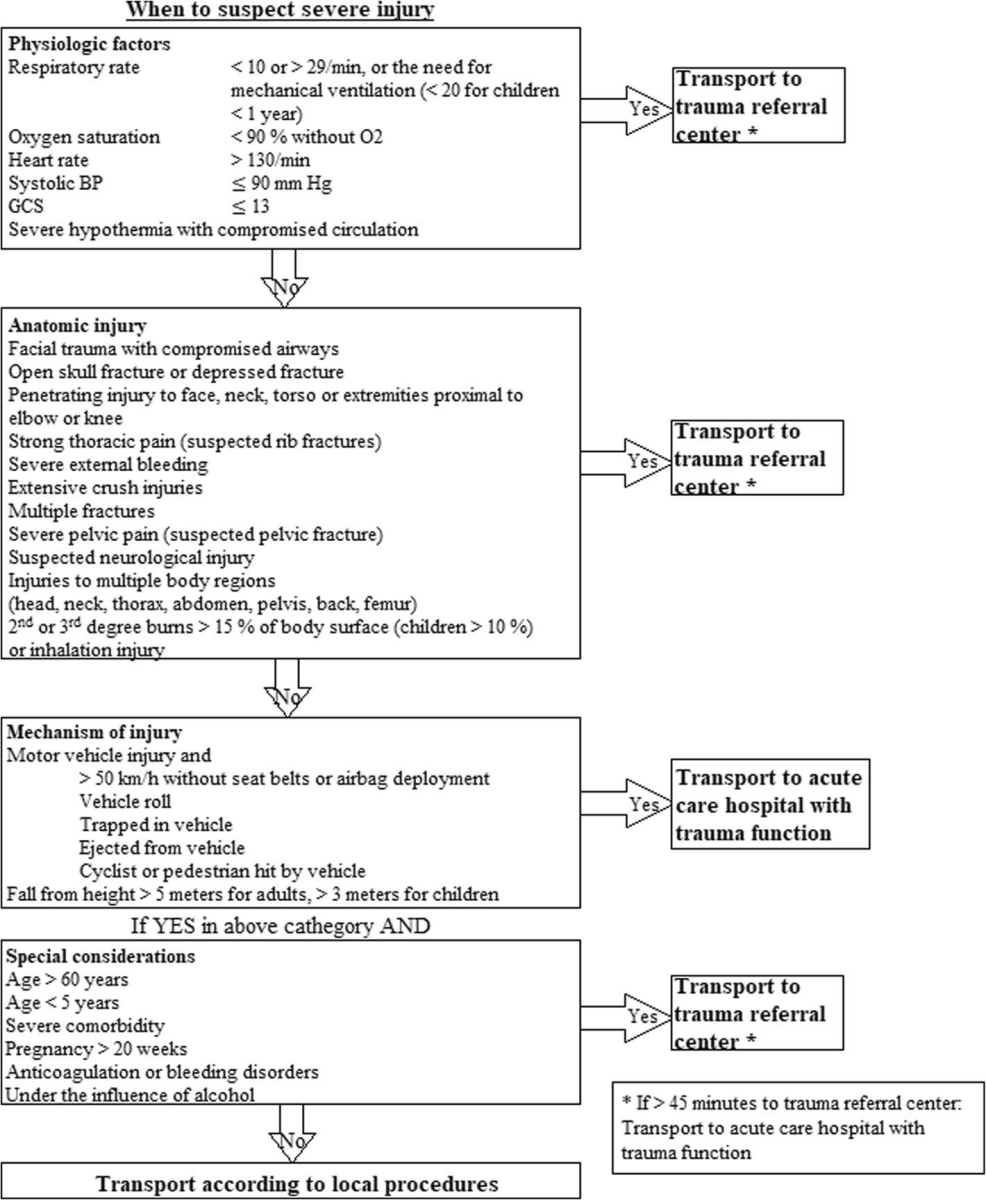
Transport criteria

All the Regional Health Thrusts in Norway have recently implemented the “*National Trauma Plan 2016”*, a national protocol for managing trauma patients. The new trauma plan recommends the use of a modified version of the “*Guidelines for Field Triage of Injured Patients*” nationwide for field triage as well as in hospitals, including pediatric trauma (Figs. 1 and 2) [19]. The guidelines recommend a four-stage triage process based on deviations in vital signs, anatomical injury, mechanism of injury (MOI) and special considerations, in descending priority. Patients who fail to meet the physiological criteria should be evaluated in terms of anatomical injury, then in terms of mechanism of injury, and so on. The decision scheme is widely implemented in the US health care system [20, 21], has been regularly revised since its inception in 1976, and underwent its latest update in 2011 [20]. Several studies have found the tool to be highly sensitive for identifying severe trauma (> 95%) [22, 23]. However, some studies indicate a lower sensitivity than previously thought [24], especially among elderly patients [25–28].

The Norwegian health system is divided into four health thrusts. Each thrust has a regional trauma center in addition to acute care hospitals with trauma function. Trauma centers provide definite care for all injuries and have access to all surgical specialties. Our study was conducted at Haukeland University Hospital (HUH). HUH is the local hospital for a population of 380,000 inhabitants and also serves as a regional trauma center for 1.1 million people in Hordaland, Rogaland and Sogn og Fjordane counties [29]. This region consists of five acute care hospitals, with HUH functioning as the regional trauma center in Western Norway. The national burn unit in Norway is located at HUH. HUH admits approximately 350–400 trauma patients each year, of which 75–90 have ISS ≥ 15.

Trauma team activation at HUH is based on initial pre-hospital information or on in-hospital clinical assessment. Single-tiered trauma team is used regardless of the assumed degree of injury. Until 2016, HUH used local criteria for trauma team activation. These criteria were mainly centered on anatomical injury, MOI and, to a lesser extent, vital signs. Despite undertriage being acknowledged as a useful tool when assessing the quality of a trauma system, no systematic investigations of triage accuracy have previously been conducted at HUH. The purpose of this study was twofold: to investigate the rate of undertriage at HUH, and to evaluate the ability of the modified version of the “*Guidelines for Field Triage of Injured Patients”* to identify severely injured patients.

## Methods

Our retrospective study included 30,444 patients admitted to HUH in 2013. Data were obtained from HUH’s patient registry, which entails information about all patients who have received specialized health care services. Patient identity was anonymized. The data set was based on discharge codes according to the 10th version of the International Statistical Classification of Diseases and Related Health Problems (ICD-10). The patients in the data set had codes ranging from S00 to T88.

Injury Severity Score (ISS) is a well-established scoring system for multi trauma, used to determine injury severity and risk of mortality [30]. Each injury is categorized according to the Abbreviated Injury Scale (AIS) [31]. ISS is the sum of squares from the highest AIS grades in the three most severely injured ISS body regions (see Appendix for further details).

**Fig. 3:**
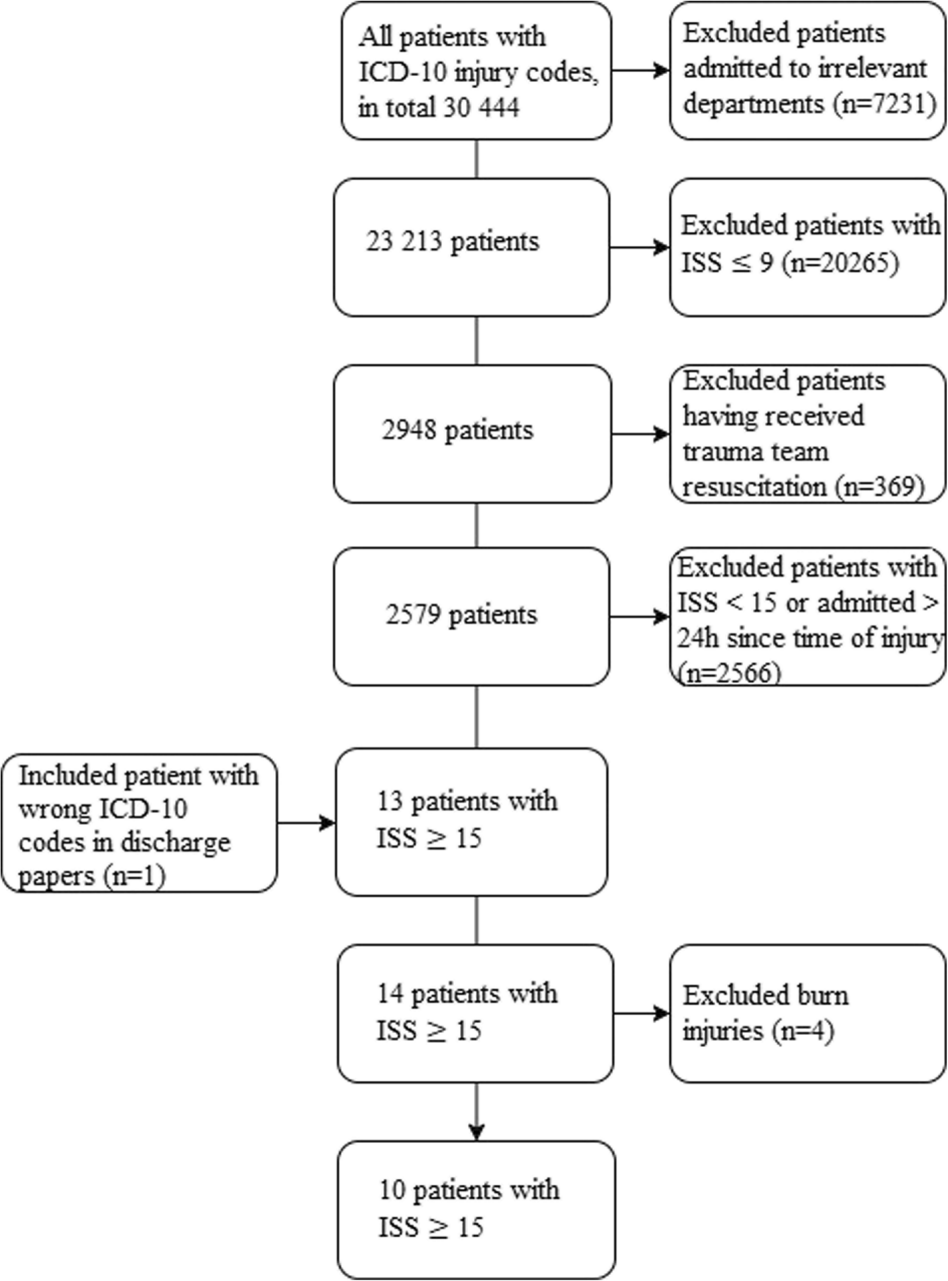
Methods

In order to find potentially undertriaged patients among the 30,444 admissions, we excluded patients admitted to departments considered unlikely to handle trauma (Table 1) and patients with single injuries where the AIS score was ≤3 (Table 2). Patients with multiple injuries in the same body region where the highest injury gave AIS ≤ 3 and where there were no injuries to other body regions, were also excluded (Table 3). Lastly, we excluded patients registered as trauma team recipients. This process was done by using filtration in Microsoft Excel and the local trauma register (see Appendix for details). Following this, 2579 medical records were manually reviewed by the first and second author. We now excluded patients with ISS < 15 or admitted > 24 h after time of injury. Every patient with ISS ≥ 13 was double-checked by the last author. One patient with ISS ≥ 15 was already registered as undertriaged in the local trauma register but was not identified in our filtration due to incorrect ICD-10 coding upon discharge (lack of S or T codes). Burn patients were excluded. The reason for this is that patients with isolated burn injuries were not routinely considered in need of trauma team according to practice in 2013. They were instead handled by a dedicated team from the burn unit. The remaining patients had ISS ≥ 15, were admitted < 24 h after time of injury and not met by trauma team (Fig. 3).

**Table 1:** Included and excluded departments

Included departments	Excluded departments
Dep. of Orthopedic Surgery	Dep. for sexually transmitted diseases
Dep. of Internal Medicine	Dep. of Rehabilitation
Dep. of Plastic and Reconstructive Surgery	Dep. of Breast and Endocrine Surgery
Dep. of Neurology	Dep. of immunology and transfusion medicine
Dep. of Thoracic Surgery	Dep. of Oncology and Medical Physics
Dep. of Gastric Surgery	Dep. of Foreign treatment
Dep. of Otorhinolaryngology	Dep. of Physiotherapy
The Burn Unit	Dep. of Habilitation services for adults
Dep. of Ophthalmology	Dep. of Dermatology
Dep. of Pulmonology and Respiratory Medicine	Kysthospitalet i Hagevik
Dep. of Vascular Surgery	Dep. of Orthopedic Rehabilitation
Dep. of Pediatrics	Dep. of Rheumatology
The Department of Anesthesiology, Perioperative and Pain Medicine	Dep. of Palliative Care
Dep. of Neurological Surgery	Dep. of Occupational Medicine
Dep. of Obstetrics and Gynecology	Voss Delivery Ward
Dep. of Thoracic Surgery	Voss Gynecology Ward
Dep. of Oral and Maxillofacial Surgery	Voss Medical Ward
Dep. of Urologic Surgery	Voss Dep. of Physiotherapy

**Table 2:** Excluded single injury ICD-10 codes

S-Codes								
Injuries to the head	S00	S022	S023	S025	S026	S028	S03	S04
	S05	S06	S08	S09				
Injuries to the neck	S10	S16						
Injuries to the thorax	S20	S223						
Injuries to the abdomen, lower back, lumbar spine, pelvis and external genitals	S30							
Injuries to shoulder and upper arm	S40	S41	S42	S43	S44	S45	S46	
Injuries to elbow and forearm	S50	S51	S54	S56	S57	S59		
Injuries to the wrist, hand and fingers	S60	S61	S62	S63	S64	S66		
Injuries to hip and thigh	S70	S72	S73	S74	S76			
Injuries to knee and lower leg	S80	S81	S82	S83	S84	S85	S86	
Injuries to ankle and foot	S90	S91	S92	S93	S94	S95	S96	S98
T-Codes								
Injuries involving multiple body regions	T00							
Effects of foreign body entering through natural orifice	T15	T16	T17	T18	T19			
Burns and corrosions of external body surface, specified by site	T23	T25						
Burns and corrosions confined to eye and internal organs	T26							
Burns and corrosions of multiple and unspecified body regions	T301							
Poisoning by, adverse effect of and under dosing of drugs, medicaments and biological substances	T4n	T41	T50					
Toxic effects of substances chiefly nonmedicinal as to source	T51	T52	T53	T54	T55	T56	T57	T58
	T59	T60	T61	T62	T63	T64	T65	
Other and unspecified effects of external causes	T66	T67	T68	T69	T70	T71	T72	T73
	T74	T75	T76	T77	T78			
Complications of surgical and medical care, not elsewhere classified	T80	T81	T82	T83	T84	T85	T86	T87
	T88							
Complications after injury, poisoning and other consequences of external injury	T90	T91	T92	T93	T94	T95	T96	T97
	T98							

Any patient with these S- and T-codes as its only injury code, were excluded. Single injuries with AIS ≤ 3 and would not result in ISS > 9. ICD-10 T40–78 and T-80-99 were excluded since they are not a direct consequence of trauma

**Table 3:** Excluded multiple injury ICD-10 codes

Injuries to the head	S00	S022	S023	S025	S026	S03	S04	S05
	S08	S09						
Injuries to shoulder and upper arm	S40	S42	S43	S44	S45	S46		
Injuries to elbow and forearm and injuries to the wrist, hand and fingers	S50	S51	S52	S53	S54	S55	S56	S57
	S60	S61	S62	S63	S64	S66	S67	S69
Injuries to knee and lower leg and injuries to ankle and foot	S80	S81	S82	S83	S84	S86		
	S90	S91	S92	S93	S94	S95	S96	S98

Patients with multiple injuries located in the same ISS body region, but with no injuries located in other regions, were excluded if the injuries included these ICD-10 codes. When receiving multiple injures with AIS ≤ 3 in the same body region, only one injury from every region is included when calculating ISS

Sensitivity and undertriage was defined in the same way as in another Norwegian study: Sensitivity as the proportion of severely injured patients managed by trauma team, and undertriage as the probability of a severely injured patient not receiving trauma team resuscitation during admission (i.e. 1-Sensitivity) [32]. The guidelines’ sensitivity was assessed by acquiring vital signs, anatomical injury and MOI from emergency department journals and emergency medical technician journals from every severely injured patient. We did not investigate the decision scheme’s specificity as this was considered beyond the scope of our study.

Informed consent was not required, as undertriage is part of the trauma system quality assessment. Approvals from the Regional Committee for Research Ethics and the Data Protection Official were waived (ref**.** no. **2015/259)**.

## Results

In 2013, 85 of the 369 patients who were met by trauma team were severely injured (ISS ≥ 15). Ten severely injured patients did not receive trauma team resuscitation. In total, 95 patients qualifying for trauma team activation were admitted (85 + 10). This gives an undertriage of 10.5% (1–85/95 or 10/95) (see Table 4).

**Table 4:** Undertriage calculation

	Severely injured	Not severely injured	Total
Trauma team activation	85	284	369
No trauma team activation	10	30,065	30,075
Total	95	30,349	30,444

Sensitivity: 85/95

Undertriage: 1 – Sensitivity = 1–85/95 or 10/95 = 10.5%

A further look into the characteristics among the undertriaged patients disclosed the following: Nearly all (nine) were men, with a median age of 54 years and median ISS of 16. Half of the patients were 60 years or older. With regard to the mechanism of injury, all of the patients experienced blunt trauma by fall from low heights (< 4 m). Eight patients had injuries located to a single anatomical region, while two patients had ≥2 body regions affected. A majority (seven) experienced injuries to head or neck.

Six patients were diagnosed with traumatic brain injury (TBI) (See Appendix for definition). Six patients had Glasgow Coma Scale score (GCS) ≤ 13, either in- or out of hospital. The patients’ Glasgow Outcome Score (GOS) (see Appendix) ranged between 1 and 5. Seven patients scored 5, one scored 4. The remaining two patients died (GOS 1). The deceased were aged 79 and 86, with ISS 26 and 25 respectively, and both died from injuries to a single body region. See Table 5 for further details.

**Table 5:** Patient information

Patient	Age	Sex	Multi trauma	MOI	Injured body region	Pre-hospital GCS	In-hospital Systolic BP	In-hospital Diastolic BP	In-hospital Pulse	In-Hospital Respiratory Frequency	In-hospital GCS	ISS	GOS	Death
1	8	Male	No	Fall in children’s slide	Head	Data missing	102	54	78	20	15	16	5	No
2	20	Male	No	Fall in staircase	Abdomen	Data missing	137	87	92	16	15	16	5	No
3	30	Male	No	Fall from installation art, 2 m	Head	Data missing	107	55	76	20	13	16	5	No
4	31	Male	No	Presumed ground level fall.	Head	13	113	70	67	20	13	26	5	No
5	48	Male	Yes	Ground level fall	Head Thorax Extremity	13	113	70	69	20	13	16	5	No
6	60	Male	No	Fall from horseback	Abdomen	15	127	70	74	19	15	16	5	No
7	67	Male	Yes	Fall from ladder, 2–3 m	Thorax Extremity	15	111	62	75	19	15	17	5	No
8	79	Male	No	Fall in staircase	Head	11	162	82	75	22	7	26	1	Yes
9	86	Male	No	Ground level fall	Neck	11	90	50	40	12	3	25	1	Yes
10	70	Female	No	Ground level fall	Head	8	150	80	80	15	7	16	4	No

We retrospectively applied the Norwegian “*Guidelines for Field Triage of Injured Patients”* as stated in the national trauma plan, on all the severely injured trauma patients in 2013 (patients with ISS ≥ 15, both undertriaged and correctly triaged) to evaluate the guidelines’ ability to identify severe trauma. Out of the 95 severely injured patients, the decision scheme identified 90 patients, showing a sensitivity of 95.0%. Deviation in vital signs was the highest-ranking criterion in 65 patients (68.0%). Among the undertriaged patients, six out of ten could have been identified as severe trauma based on vital sign deviation (reduced GCS). Anatomical injuries were the highest-ranking criterion in 21 patients (22.0%). Four (4.0%) were identified from MOI alone. None of the undertriaged patients could have been identified based on anatomic injury or MOI alone. By retrospectively applying the guidelines to the 2013 patients, the undertriage was reduced from 10.5 to 4.0%.

## Discussion

Retrospective data from 2013 indicate 10.5% undertriage among trauma patients at HUH. Our data imply a rate of undertriage at HUH which is more than twice as high as national recommended benchmarks [19]. Scandinavian studies have reported similar or higher rates at other Trauma Centers [32–35]. Studies from American Emergency Departments have found even higher undertriage, ranging from 40 to 70% [27, 36], demonstrating that assessing injury severity remains a significant challenge.

The high rate of undertriage among elderly trauma patients is also described elsewhere [24–28, 37, 38], including in studies using “*Field Triage of Injured Patients”* [25, 28]. Our data shows a skewness towards high age, but the small sample size prevents us from making any firm conclusions. Moreover, some have pointed out that vital signs are less reliable to predict injury severity among patients > 65 years of age [37, 39]. Other studies have found increased mortality risk among elderly patients after ground level falls [40, 41]. Such MOI is not severe enough to activate trauma team, and the high-risk patients should therefore be identified by different means. Additionally, elderly injured patients raise unique challenges, such as potential for higher degree of comorbidity, use of anticoagulants, higher operative risk, and secondary medical complications. As a consequence, the trauma related mortality is higher in the geriatric population [42, 43]. To counter this, high age alone has been suggested as a criterion for trauma team activation [44, 45]. This is not current triage practice in Norway [19]. Still, age > 60 years is a criterion under “*Special considerations”* which should lower the threshold for trauma team activation and referral to regional trauma center if transport time < 45 min. Awareness of the special circumstances related to this patient group might aid the triage process and possibly reduce trauma mortality.

Several studies have found that undertriage regularly affects patients with head injury [24, 27, 33]. Xiang et al. reported that > 40% of the undertriaged trauma patient diagnosis were TBIs (See Appendix for definition). Our data showed the same trend, as more than half of the patients (6/10) were diagnosed with TBI. TBI leads to increased mortality and permanent disabilities [46–50], making early access to proper care crucial.

Vital signs have been proven useful when identifying severe trauma [32, 51–53] and GCS has shown to be a good predictor of mortality [54–56]. Pearson and colleagues found that patients with TBI (See Appendix for definition) and a GCS score ≤ 13 were 17 times more likely to die compared to those with a higher GCS score, after controlling for age, gender, race, ISS and length of hospital stay [57]. Others have criticized GCS for its poor ability to predict isolated head injuries among older trauma patients [58].

Our findings suggest that the “*Guidelines for Field Triage of Injured Patients*” have a higher sensitivity (95%) than indicated by recent studies [25, 28]. The lowest sensitivity (66%) was reported in a prospective study from 2016 including 53,487 patients [28]. A contributing factor behind this discrepancy could be that our study population was sampled from a single regional trauma center only, excluding acute care hospitals in our health region. We are unable to determine to what extent severely injured patients were incorrectly transported to acute care hospitals with trauma function, without access to the relevant care. This means that our study cannot appraise the guidelines’ ability to identify patients suitable for direct transport or transfer to a regional trauma center, only their ability to identify in-hospital severe trauma and need for trauma team activation. Consequently, we can neither confirm nor disconfirm the findings of recent studies suggesting that transport to lower tiered hospitals (both trauma and non-trauma hospitals) contributes to the guidelines’ low sensitivity [25, 28].

Although the “*Guidelines for Field Triage of Injured Patients*” had a high total sensitivity (95.0%), only 68.0% of the severely injured patients were identified based on vital signs alterations, proving that vital signs alone were insufficient to identify severe trauma. However, the sensitivity improved substantially by combining vital sign deviations with defined anatomical injuries. Cook and colleges hypothesized that the use of vital signs and anatomical injury alone might be sufficient to identify need of trauma team [59]. MOI alone was able to identify only a small portion of severely injured patients. However, MOI inclusion was required to achieve the desired 95% sensitivity, which is in line with what prior studies have indicated. Based on our limited data, we recommend adherence to the new guidelines and propose implementing simple tools such as checklists to use both out-of- and in-hospital.

There are several limitations in our study. The most important one is the low sample size, due to both short study length, low trauma volume and the amount of severe trauma admitted to HUH per year. The findings should therefore be interpreted cautiously, bearing in mind the possible implications low sample size may have for their representativeness. Additionally, only patients admitted to a single trauma referral center was included. It is therefore possible that regional characteristics have influenced our trauma population. Only ISS score was used to evaluate trauma severity. This is recommended by both the national trauma plan and American College of Surgeons in quality assessment [18, 19], while being challenged by others for its low ability to predict outcome compared to other trauma scoring systems [60–64]. Patient injuries were recorded using ICD-10 codes. Given the possibility of erroneous coding in the discharge papers, some undertriaged patients may have been missed. Lastly, each injury was assigned the closest corresponding AIS-code, a procedure that could reduce the accuracy of the individual injury descriptions.

## Conclusion

Undertriage at HUH was 10.5%. Among the undertriaged, elderly patients with low level falls and subsequently isolated head and neck injuries dominated. With correct use of the modified version of “*Guidelines for Field Triage of Injured Patients*” the rate of undertriage could have been reduced by more than 50%, thereby keeping in line with the recommended < 5% undertriage. Our data indicate that the guidelines have a high sensitivity when identifying severely injured patients in need of trauma team activation.

## Appendix

### AIS and ISS calculation

Abbreviated Injury Scale is an anatomical-based coding system. As explained in the AIS 2005 manual (update 2008) each injury is assigned a unique seven-digit code describing type, location and severity.

AIS grades the severity of each injury in the following manner:

0 – No injury.
1 – Minor.
2 – Moderate.
3 – Severe (not life-threatening).
4 – Severe (life threatening, survival probable).
5 – Severe (critical, survival uncertain).
6 – Maximal, possibly fatal.

In order to calculate the Injury Severity Score (ISS) the body is divided into six body regions. The manual describes ISS as the sum of squares from the highest AIS grades in the three most severely injured ISS body regions.

$$ ISS={A}^2+{B}^2+{C}^2 $$

A, B and C are the AIS scores from the three most severely injured ISS body regions. The ISS score ranges from 1 to 75. Any injury assigned an AIS 6 leads to an ISS 75.

### Excel filtration

In order to uncover every severely injured trauma patient, we used four sets of exclusion criteria in our filtration process. First, departments considered unlikely to admit severe trauma were excluded (Table 1). Secondly, patients with single injury ICD-10 codes resulting in AIS ≤ 3 were excluded. Thirdly, patients with multiple injuries in the same body region where the highest injury gave AIS ≤ 3 and where there were no injuries to other body regions were excluded. Lastly, patients identified as being met by trauma team were excluded using HUH’s local trauma register. The filtering was done by using advanced filtering functions in Microsoft Excel 2013 (v15.0). This process excluded a total of 27,865 patients. The remaining patients were manually reviewed.

2 × 2 Contingency table for defining undertriage.

**Table:**

	Severely injured	Not severely injured	Total
Trauma team activation	(a)	(b)	(a + b)
No trauma team activation	(c)	(d)	(c + d)
Total	(a + c)	(b + d)	(n)

$$ Sensitivity=a/\left(a+c\right) $$

$$ Undertriage=1- Sensitivity=c/\left(a+c\right) $$

### Traumatic Brain Injury definition

We defined TBI with reference to the following ICD-10 codes: S06.0-S06.6.

Xiang and colleges defined TB with reference the following ICD-9 codes: 800.0–801.9, 803.0–804.9, 850.0–854.0, and 959.01, excluding 995.55.

Pearson and colleges defined TBI with reference to the following ICD-9 codes: 800.0–801.9, 803.0–804.9, 850.0–854,1, 950.1–950.3, 959.01.

Glasgow Outcome Score.

**Table:**

GOS score	Functional status
5	Resumption of normal life, possibly minor neurological and/or psychological deficits
4	No need for daily support but may require special adjustments at work.
3	Permanent need for help with daily living
2	Prolonged state of unresponsiveness for weeks, months or until death
1	Death

## Abbreviations

AIS: Abbreviated Injury Scale
GCS: Glasgow Coma Scale
GOS: Glasgow Outcome Score
HUH: Haukeland University Hospital
ICD-10: International Classification of Disease, 10th version
ICD-9: International Classification of Diseases, 9th version
ISS: Injury Severity Score
MOI: Mechanism of Injury
TBI: Traumatic Brain Injury
